# Induction of Antinuclear Antibodies by De Novo Autoimmune Hepatitis Regulates Alloimmune Responses in Rat Liver Transplantation

**DOI:** 10.1155/2013/413928

**Published:** 2013-12-23

**Authors:** Toshiaki Nakano, Shigeru Goto, Chia-Yun Lai, Li-Wen Hsu, Hui-Peng Tseng, Kuang-Den Chen, King-Wah Chiu, Chih-Chi Wang, Yu-Fan Cheng, Chao-Long Chen

**Affiliations:** ^1^Graduate Institute of Clinical Medical Sciences, Chang Gung University College of Medicine, 123 Ta-Pei Road, Niao-Sung, Kaohsiung 833, Taiwan; ^2^Liver Transplantation Program, Center for Translational Research in Biomedical Sciences, Kaohsiung Chang Gung Memorial Hospital and Chang Gung University College of Medicine, 123 Ta-Pei Road, Niao-Sung, Kaohsiung 833, Taiwan; ^3^Division of Transplant Immunology, Center for Translational Research in Biomedical Sciences, Kaohsiung Chang Gung Memorial Hospital and Chang Gung University College of Medicine, 123 Ta-Pei Road, Niao-Sung, Kaohsiung 833, Taiwan; ^4^Iwao Hospital, 3059-1 Kawakami, Yufu, Oita 879-5102, Japan; ^5^Department of Veterinary Medicine, National Pingtung University of Science & Technology, 1 Shuehfu Road, Neipu, Pingtung 912, Taiwan

## Abstract

Concanavalin A (Con A) is a lectin originating from the jack-bean and well known for its ability to stimulate T cells and induce autoimmune hepatitis. We previously demonstrated the induction of immunosuppressive antinuclear autoantibody in the course of Con A-induced transient autoimmune hepatitis. This study aimed to clarify the effects of Con A-induced hepatitis on liver allograft rejection and acceptance. In this study, we observed the unique phenomenon that the induction of transient de novo autoimmune hepatitis by Con A injection paradoxically overcomes the rejection without any immunosuppressive drug and exhibits significantly prolonged survival after orthotopic liver transplantation (OLT). Significantly increased titers of anti-nuclear Abs against histone H1 and high-mobility group box 1 (HMGB1) and reduced donor specific alloantibody response were observed in Con A-injected recipients. Induction of Foxp3 and IL-10 in OLT livers of Con A-injected recipients suggested the involvement of regulatory T cells in this unique phenomenon. Our present data suggest the significance of autoimmune responses against nuclear histone H1 and HMGB1 for competing allogeneic immune responses, resulting in the acceptance of liver allografts in experimental liver transplantation.

## 1. Introduction

Liver is permanently exposed to gut-derived antigens, including pathogens, toxins, and harmless food antigens, and immune responses against the dietary or bacterial antigens from the gut are unusual [1, 2]. However, the immune system should be activated to prevent liver damage when the liver is suffering from harmful pathogens such as hepatitis B and C viruses. The mechanisms for balancing tolerance and immunity in the liver have not been fully elucidated, but the unique repertories of nonparenchymal cells including liver antigen-presenting cells (e.g., dendritic cells (DCs), Kupffer cells, and liver sinusoidal endothelial cells) and unconventional lymphoid cells (e.g., NK cells, B-1 cells, and *γδ* T cells) that are rarely present in the blood may explain the immune privilege of the liver [3]. Furthermore, it has been known that the liver does not always obey the normal rules of transplant rejection (Medawar's rule of transplantation) [4].

In a rat orthotopic liver transplantation (OLT) model, Piebald Virol Glaxo (PVG) (MHC haplotype; RT1^c^) recipients spontaneously accept donor Dark Agouti (DA) (RT1^a^) livers in the absence of extra immunosuppressive treatment, while other organ allografts in this combination are promptly rejected [5–7]. In contrast, recipient Lewis (LEW) (RT1^l^) rats usually reject a donor DA rat liver within 14 days after OLT [8–10]. In our previous studies, we have compared the serum protein profiling in these OLT models (tolerogenic DA-PVG versus rejecting DA-LEW) and demonstrated the spontaneous induction of autoantibody (auto-Ab) against nuclear histone H1 and high-mobility group box 1 protein (HMGB1) in the DA-PVG natural tolerance model [11–14]. Further research has demonstrated that the survival of heart allografts into histone H1-immunized rats was significantly prolonged along with the elevation of the anti-histone H1 Ab titer in the DA-LEW rejection combination [15, 16]. In addition to the involvement of Ab response against nuclear histone H1 in liver transplant tolerogenicity [17], anti-histone H1 Ab was found to be expressed spontaneously in sera during the recovery stage from Concanavalin A (Con A-) induced transient liver injury, suggesting the significance of anti-histone H1 Ab as a regulatory Ab (Abreg) for both protection and recovery from autoimmune hepatitis [18].

Autoimmune hepatitis is characterized by a chronic inflammatory disease with elevation of serum auto-Ab (e.g., antinuclear Ab, smooth muscle Ab, and liver-kidney microsome Ab), with hypergammaglobulinemia and liver pathology showing necroinflammatory disease and fibrosis [19, 20]. Currently, the only viable treatments for autoimmune hepatitis are immunosuppressant application and liver transplantation. In addition, the development of newly created (de novo) autoimmune hepatitis has been reported after liver transplantation since 1998 [21]. Patients who develop de novo autoimmune hepatitis do not have a satisfactory response to standard antirejection regimens, but they do respond to the standard treatment for autoimmune hepatitis (steroids and azathioprine) in combination with a low dose of calcineurin inhibitor [22]. However, little is known about the immunological aspects of de novo autoimmune hepatitis on liver allograft rejection and acceptance in liver transplantation.

In this study, we performed OLT in the rejection combination (DA-LEW) and then induced transient de novo autoimmune hepatitis by Con A injection three days after OLT to evaluate the effects of transient de novo autoimmune hepatitis on liver allograft survival and immune responses.

## 2. Materials and Methods

### 2.1. Animals

Male DA (RT1^a^) and LEW (RT1^l^) rats at 4 weeks of age were obtained from Japan SLC (Hamamatsu, Japan) and the National Laboratory Animal Breeding and Research Center (Taipei, Taiwan), respectively. All animals were maintained in special pathogen-free animal facilities with water and commercial rat food provided *ad libitum*.

Our experimental design was reviewed and approved by the Institutional Animal Care and Use Committee, and the committee recognizes that the proposed animal experiment follows the Animal Protection Law by the Council of Agriculture, Executive Yuan, Taiwan, and the guideline as shown in the Guide for the Care and Use of Laboratory Animals as promulgated by the Institute of Laboratory Animal Resources, National Research Council, USA.

### 2.2. Orthotopic Liver Transplantation (OLT) and the Induction of De Novo Autoimmune Hepatitis

OLT was carried out following the technique previously described by Kamada and Calne [23] in the DA to LEW (DA-LEW) combination, which is known as an acute rejection model. All serum samples were stored at −80°C until analysis.

To induce de novo autoimmune hepatitis, 20 mg/kg of Con A (type IV) (GE Healthcare Bio-Sciences Corp., Piscataway, NJ, USA) dissolved in PBS was administered intravenously via the penile vein [18, 24] into the recipient LEW rats at day 3 after OLT or into the naïve LEW rats. In the control group, PBS was injected on the same schedule as the Con A group.

### 2.3. Histological Evaluation

Liver tissues were taken at postoperative day 7 (rejection phase) from the DA-LEW OLT rats with PBS or Con A injection, fixed in formaldehyde and embedded in paraffin wax prior to sectioning. In addition, liver tissues from long surviving rats were taken at >200 days after OLT from DA-LEW OLT rats with Con A injection. Liver tissues from naïve DA rats were used as a control. For histological evaluation, paraffin sections (3 *μ*m thick) were heated for 15 min at 56°C, deparaffinized, rehydrated, and stained with hematoxylin (Merck KGaA, Darmstadt, Germany) and eosin (Sigma, St. Louis, MO, USA) according to the manufacturer's protocol. All sections were examined using a light microscope (Olympus, Tokyo, Japan).

### 2.4. Circulating Levels of Nuclear Histone H1 and HMGB1

The levels of serum histone H1 and HMGB1 were determined by enzyme-linked immunosorbent assay (ELISA) as described previously [25]. For the quantitative determination of histone H1, 0.1 *μ*g of anti-histone H1 polyclonal Ab (Santa Cruz Biotechnology, Santa Cruz, CA, USA) in 100 mM NaHCO_3_ (pH 9.3) was coated onto a 96-well microtiter plate (Nalge Nunc International, Roskilde, Denmark) by overnight incubation at 4°C. The plate was then blocked with SuperBlock T20 (PBS) Blocking Buffer (Thermo Fisher Scientific Inc., Rockford, IL, USA), and serum samples (50 *μ*L, ×25 dilution with 10 mM Tris-HCl (pH 8.0), 0.9% (w/v) NaCl, 0.5% (w/v) Tween 20) were added to the wells. Calf thymus histone H1 (Upstate, Charlottesville, VA, USA) was used as a standard. The mixture was incubated at room temperature for 1 hr. Anti-histone H1 monoclonal Ab (×500 dilution; Abcam, Cambridge, MA, USA) was then added, and the mixture was incubated at room temperature for 1 hr. Peroxidase-conjugated anti-mouse IgG (×2,000 dilution; Santa Cruz Biotechnology) was then added, and the mixture was incubated at room temperature for 1 hr, followed by the addition of 1-Step Ultra TMB substrate solution (Thermo Fisher Scientific Inc.). For the quantitative determination of HMGB1, a rat HMGB1 ELISA kit (MyBioSource Inc., San Diego, CA, USA) was used according to the manufacturer's protocol. The absorbance (450 nm) was then measured using a Victor X4 Multilabel Plate Reader (PerkinElmer, Shelton, CT, USA).

### 2.5. Measurement of Anti-Histone H1 and HMGB1 Ab Titer by ELISA

We used an ELISA to evaluate the anti-histone H1 and HMGB1 Ab titers as described previously [11–14] with minor modifications. To evaluate the anti-histone H1 and HMGB1 Ab titers, 2 *μ*g/mL of calf thymus histone H1 (Upstate) or recombinant human HMGB1 (Sigma) in 100 mM NaHCO_3_ (pH 9.3) was coated onto a 96-well microtiter plate (Nalge Nunc International) by incubation at room temperature for 1 hr. The plate was then blocked with SuperBlock T20 (PBS) Blocking Buffer (Thermo Fisher Scientific Inc.), and serum samples (50 *μ*L, ×100 dilution with 10 mM Tris-HCl (pH 8.0), 0.9% (w/v) NaCl, 0.5% (w/v) Tween 20) from the recipient LEW rats after OLT or nontransplanted LEW rats with Con A injection were added to the wells. The mixture was incubated at room temperature for 1 hr. Secondary peroxidase-conjugated anti-rat IgG (×2000 dilution; Biosource International, Camarillo, CA, USA) was then added, and the mixture was incubated at room temperature for 1 hr, followed by the addition of ABTS substrate solution (Sigma). The absorbance (405 nm) was then measured using a Victor X4 Multilabel Plate Reader (PerkinElmer).

### 2.6. Measurement of Donor Specific Alloantibody (DSA) Response after OLT

The DSA response was measured by flow cytometry on a single cell suspension from DA rat splenocytes, as described previously [26] with minor modifications. To evaluate the DSA response after OLT, 50 *μ*L of aliquots containing 5 × 10^5^ splenocytes was incubated with 50 *μ*L of diluted naïve or post-OLT sera for 45 min at 4°C. The washed cells were treated with 50 *μ*L of a mixture of FITC-conjugated goat Ab specific for the Fc portion of rat IgG (×100 dilution) and PE-conjugated goat Ab specific for the *μ* chain of rat IgM (×100 dilution) (Jackson Immunoresearch Laboratories, West Grove, PA, USA) in PBS containing 1% BSA and 0.02% NaN_3_. After staining, the cells were washed, fixed, and analyzed using a LSRII flow cytometer (BD Biosciences, San Jose, CA, USA).

### 2.7. RNA Isolation and Real-Time PCR

RNA from a transplanted donor DA liver harvested at the rejection phase (postoperative day 7: *n* = 5) or a nontransplanted LEW liver at day 4 after Con A injection was extracted using TRIzol reagent (Invitrogen, Carlsbad, CA, USA) according to the manufacturer's instructions. Total RNA (2 *μ*g) was reverse-transcribed into cDNA with high capacity cDNA reverse transcription kit (Life Technologies, Carlsbad, CA, USA). The rat-specific PCR primers are as follows: GAPDH (sense), 5′-CCATGGAGAAGGCTGGGG-3′, and (antisense), 5′-CAAAGTTGTCATGGATGACC-3′; Foxp3 (sense), 5′-CCCAGGAAAGACAGCAACCTT-3′, and (antisense), 5′-CTGCTTGGCAGTGCTTGAGAA-3′; IL-10 (sense), 5′-CAGACCCACATGCTCCGAGA-3′, and (antisense), 5′-CAAGGCTTGGCAACCCAAGTA-3′. Quantitative PCR of GAPDH, Foxp3, and IL-10 was performed using a 7500 Fast Real-Time PCR System (Applied Biosystems Inc., Foster, CA, USA). The GAPDH reference gene was used to normalize the data. The 2^−Δ·CT^ value, which corresponds to the expression of each gene compared to GAPDH, and 2^−Δ·Δ·CT^, which corresponds to the expression ratio of each gene in the experimental group compared to the control, were calculated.

### 2.8. SDS-PAGE and Western Blot Analyses

To detect the protein expression of Foxp3 in livers, naïve DA livers and liver allografts at rejection (postoperative day 7) or acceptance (>200 days) were manually homogenized with T-PER Tissue Protein Extraction Reagent (Thermo Fisher Scientific Inc.) supplemented with protease inhibitor complete (Roche Diagnostics, Mannheim, Germany). After centrifugation, liver extracts (100 *μ*g) were run on a 10% sodium dodecyl sulfate-polyacrylamide gel electrophoresis (SDS-PAGE) gel using a mini gel apparatus (Bio-Rad, Burlington, MA, USA), and fractionated proteins were electronically transferred onto a polyvinylidene fluoride transfer membrane (GE Healthcare Bio-Sciences Corp.). The membrane was blocked using 5% skim milk at room temperature for 1 hr and immunoprobed with mouse monoclonal Ab against Foxp3 (×2000; Santa Cruz Biotechnology) followed by incubation with peroxidase-conjugated goat anti-mouse IgG (×10000; Santa Cruz Biotechnology). Signals were visualized using an ECL Plus Western Blotting Detection System (GE Healthcare Bio-Sciences Corp.), and relevant bands were quantified by densitometry using a G:BOX Image Station iChemi XL device (Syngene, Cambridge, UK).

### 2.9. Statistical Analysis

Unpaired two-tailed Student's *t*-tests were used to determine the significance of the difference between the normally distributed means of value in the two groups. Each sample was tested in triplicate, and the results are indicated as mean ± SD.

The actual liver allograft survival was calculated using the Kaplan-Meier product limit estimator. The log-rank test (Mantel-Cox) was used to test the equality of the graft survival curves.

## 3. Results

### 3.1. Prolongation of Allograft Survival by the Induction of De Novo Autoimmune Hepatitis

**Figure 1:**
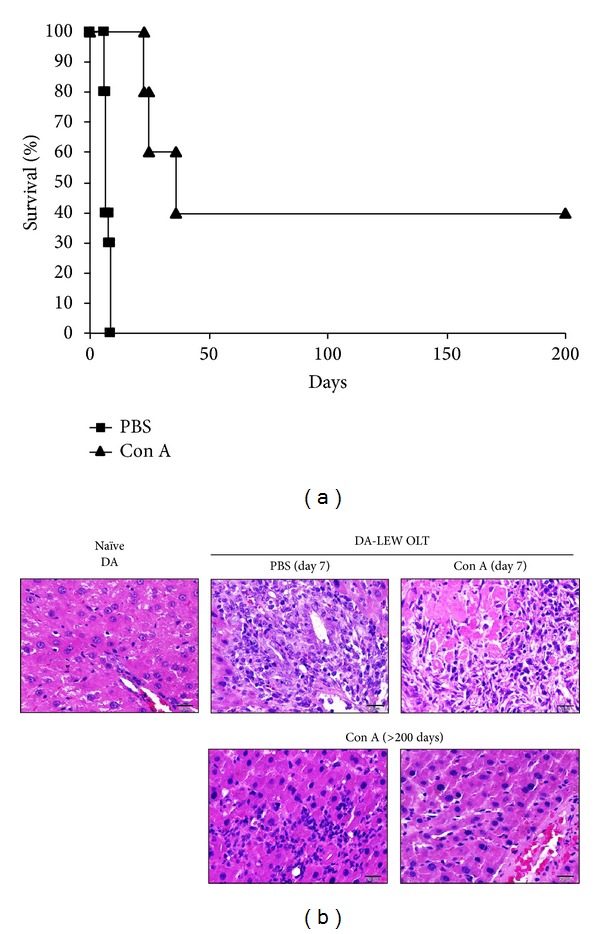
The prolongation of allograft survival by the transient induction of de novo autoimmune hepatitis. (a) The DA liver allograft survival of DA-LEW OLT with Con A injection (*n* = 5) was significantly prolonged (*P* = 0.001, log-rank test) compared with the survival in the control PBS group (*n* = 10). (b) Paraffin sections of naïve and OLT livers at rejection (day 7) or acceptance (>200 days) were stained with hematoxylin and eosin. The data are representative examples of three (naïve and OLT at day 7) or two (>200 days) individual liver sections (40x magnification, bar = 20 *μ*m).

Con A-induced acute liver injury is known as a model for autoimmune hepatitis and the peak time of hepatic inflammation is at 24 hr after Con A injection. Such hepatic inflammation is spontaneously recovered at 3 to 7 days after Con A injection with transient elevation of antinuclear Abreg against histone H1 [18]. To evaluate the effects of such transient induction of de novo autoimmune hepatitis on liver allograft survival, we performed OLT in the rejection combination (DA-LEW) and induced transient de novo autoimmune hepatitis by Con A injection three days after OLT. As shown in Figure 1(a), liver allograft survival was significantly prolonged by Con A injection. Liver histology demonstrated the massive infiltration of immune cells and damage to the hepatic parenchyma during the rejection phase (day 7) after OLT both in the control PBS and the Con A groups, while mild inflammation but not severe hepatic damage was confirmed in the liver allografts of long surviving rats (>200 days after OLT) (Figure 1(b)).

### 3.2. Suppression of Circulating Nuclear Histone H1 and HMGB1 by Con A Injection

**Figure 2:**
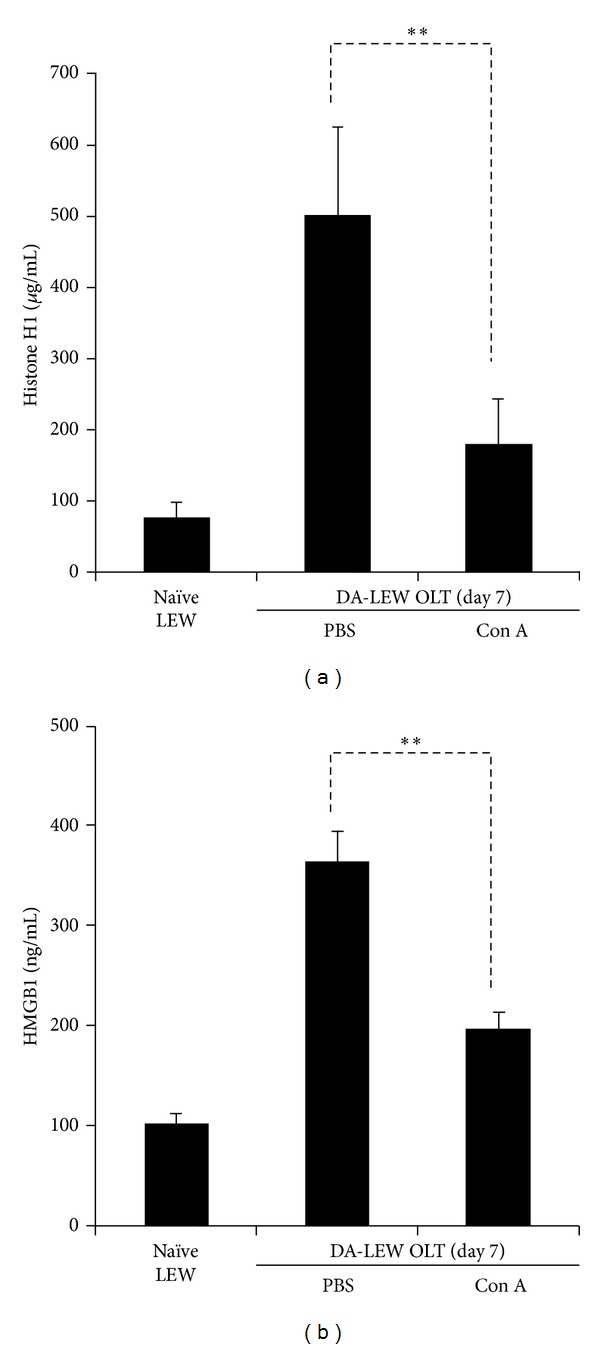
The circulating levels of nuclear histone H1 (a) and HMGB1 (b) during the rejection phase after OLT. For the quantitative determination of histone H1 and HMGB1, sandwich ELISA was performed as described in the Materials and Methods section. The results are expressed as the mean of three independent experiments ± SD. **Significantly different compared with the control PBS group (*n* = 5) (*P* < 0.01, Student's *t*-test).

The release of nuclear antigens into the blood stream has been associated with the progression of several diseases, and our previous study demonstrated the elevation of nuclear histone H1 and HMGB1 during the rejection phase (day 7) after OLT [25]. As shown in Figure 2, Con A injection after OLT significantly suppressed the circulating levels of nuclear histone H1 and HMGB1.

### 3.3. Induction of Antinuclear Antibodies after Con A Injection

**Figure 3:**
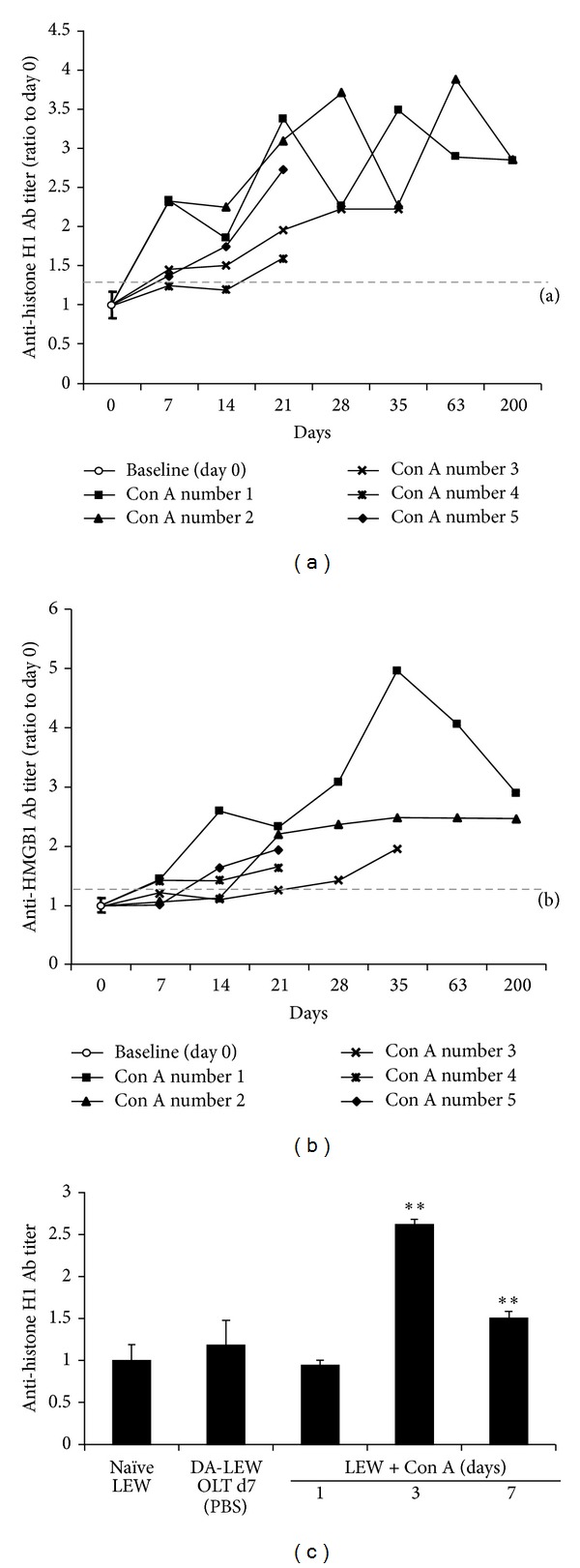
The induction of Ab against nuclear histone H1 (a) and HMGB1 (b) after OLT by the transient induction of de novo autoimmune hepatitis. Anti-histone H1 and HMGB1 Ab titers (optical density at 405 nm) were measured by ELISA as described in the Materials and Methods section. The results are expressed as the mean of three independent experiments (ratio to day 0) ± SD. The dotted line indicates cutoff values (*P* < 0.05) of 2SD above the mean of baseline (*n* = 15) (a: 1.35; b: 1.25). (c) No elevation or transient induction of anti-histone H1 Ab titer was observed in the serum of DA-LEW OLT without Con A injection or nontransplanted LEW rats with Con A injection. The results are expressed as the mean of three independent experiments (ratio to naïve LEW) ± SD. **Significantly different compared with the naïve LEW rats (*n* = 5) (*P* < 0.01, Student's *t*-test).

Anti-histone H1 and HMGB1 Abs are known as immune Abreg for overcoming rejection and subsequent liver allograft acceptance [13, 14]. To characterize the effects of antinuclear Abs on the prolongation of liver allograft survival, we next evaluated the Ab response against nuclear histone H1 and HMGB1. As shown in Figure 3(a), most (4/5) of Con A-injected recipients expressed higher titer of anti-histone H1 Ab at day 7 after OLT and remained at an elevated level compared with the baseline (day 0), while anti-histone H1 Ab was not detected in the serum of OLT (DA-LEW) without Con A treatment or transiently induced in the course of Con A-induced autoimmune hepatitis (Figure 3(c)) [13, 14, 18]. On the other hand, anti-HMGB1 Ab was upregulated at day 14 after OLT in 3/5 of Con A-injected recipients and also remained at an elevated level, similar to the anti-histone H1 Ab (Figure 3(b)). No such elevation of anti-HMGB1 Ab was observed in the control group (DA-LEW without Con A treatment) (data not shown). Notably, long surviving recipients (2/5; >200 days) showed the highest titer of both anti-histone H1 and HMGB1 Ab in the course of OLT. These results suggest the significance of Ab response against nuclear histone H1 and HMGB1 for the prolongation of allograft survival by Con A injection.

### 3.4. Suppression of Alloantibody Response by Con A Injection

**Figure 4:**
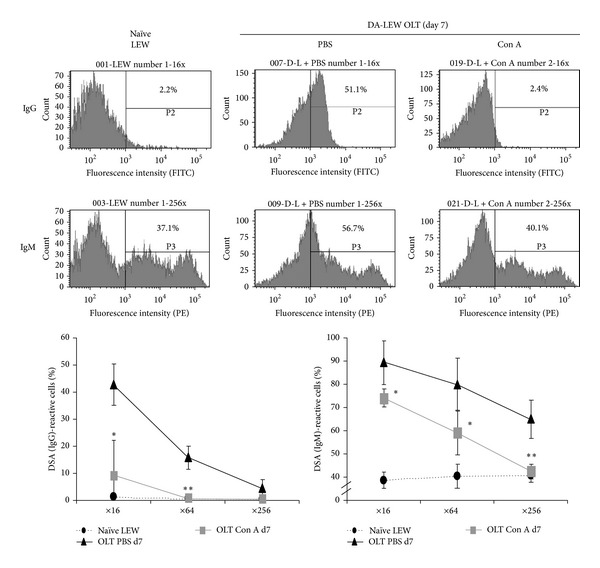
Donor specific alloantibody (DSA) response during the rejection phase after OLT. The DSA response against donor DA splenocytes was measured by flow cytometry as described in the Materials and Methods section. Histograms (representative of four individuals) show the percentage of DA splenic cells recognized by DSA (IgG and IgM) in the sera of OLT (IgG: ×16 dilution, IgM: ×256 dilution) at day 7 after OLT. *, **Significantly different compared with the control PBS group (*n* = 5) (*P* < 0.05 and 0.01, resp., Student's *t*-test).

We next evaluated whether the induction of antinuclear Abreg affects the donor specific alloantibody (DSA) response during the rejection phase (day 7) after OLT. As shown in Figure 4, the percentage of DA splenic cells recognized by DSA (IgG and IgM) in the sera of OLT without Con A injection was high but significantly reduced by Con A injection. These results suggest that the induction of Ab response against histone H1 and HMGB1 may reduce the DSA response associated with the rejection response after OLT.

### 3.5. Induction of Foxp3^+^ Regulatory T Cells in OLT Livers by Con A Injection

**Figure 5:**
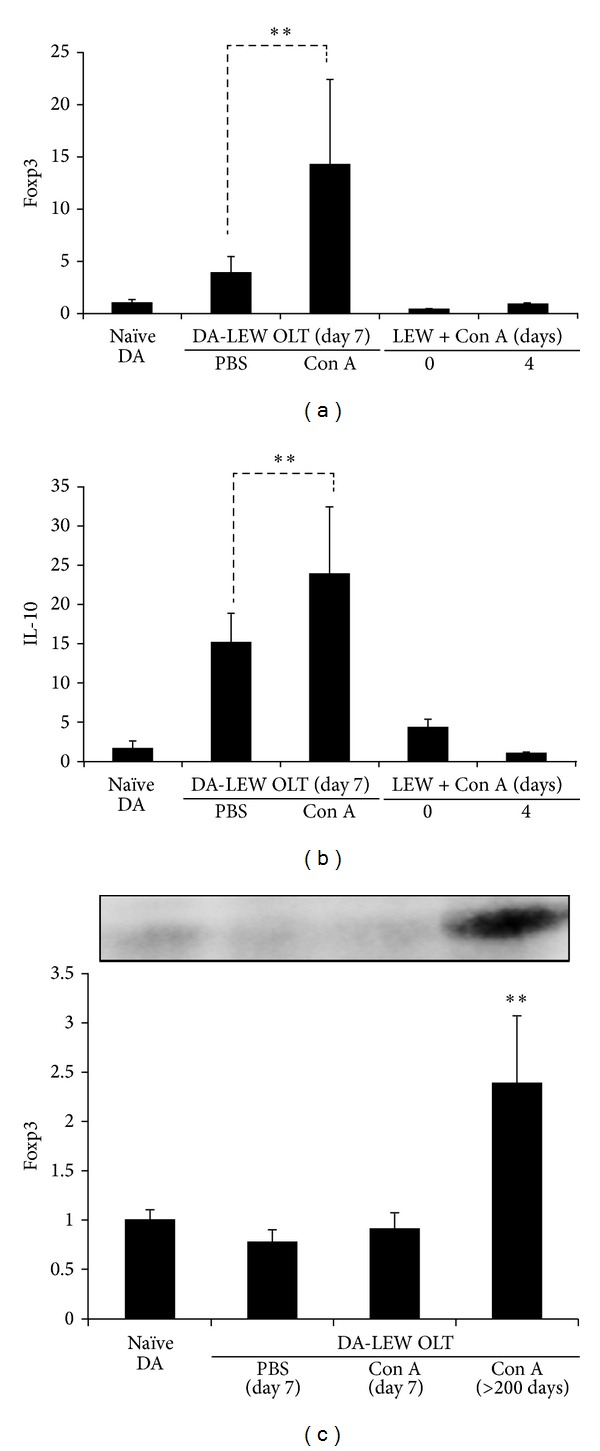
Hepatic levels of Foxp3 (a) and IL-10 (b) during the rejection phase after OLT or in the course of Con A-induced autoimmune hepatitis. The results are expressed as the mean of five individuals ± SD. **Significantly different compared with the control PBS group (*n* = 5) (*P* < 0.01, Student's *t*-test). (c) Protein level of Foxp3 in naïve and OLT livers at rejection (day 7) or acceptance (>200 days) was semiquantified by Western blot. Results are expressed as the mean of three independent experiments (ratio to naïve DA) ± SD. *Significantly different compared with naïve DA livers (*P* < 0.01).

To explore the roles of regulatory T cells (Tregs) for liver allograft prolongation in Con A-injected recipients, we next evaluated the hepatic levels of Foxp3 and the inhibitory cytokine IL-10 at rejection phase after OLT. As shown in Figure 5, we have confirmed the elevation of both Foxp3 and IL-10 in liver allografts at day 7 after OLT (i.e., at day 4 after Con A injection) while Con A injection itself was no such elevation of those factors. Hepatic level of Foxp3 protein was also increased in the long surviving liver allografts (Figure 5(c)). These results suggest the involvement of synergistic effects of autoimmune hepatitis and acute cellular rejection in liver allografts for Treg-mediated immune regulation, resulting in the long-term acceptance of liver allografts in Con A-injected recipients.

## 4. Discussion

The release of nuclear antigens and its suppression by neutralized Abs are proposed to be important in the initiation and regulation of immune responses. Wang et al. first reported the proinflammatory role of HMGB1 in endotoxin lethality in mice and in septic patients [27]. Since then, accumulating evidence has suggested the significance of HMGB1 in both innate immunity and adaptive immunity and as a therapeutic target for immune regulation [28]. The critical role of HMGB1 in the pathogenesis of Con A-induced acute liver injury was also recently demonstrated [29]. On the other hand, the roles of histones in immune responses are poorly understood in comparison with HMGB1, whereas our previous studies strongly suggested the significance of Ab response against histone H1 for overcoming rejection [11–17] and for the protection of Con A-induced acute liver injury [18]. Our subsequent study has demonstrated that the translocation of histone H1 from nucleus to cytoplasm and the release of their own histone H1 are necessary for the maturation of DCs and for the activation of T cells [30]. This function is also similar to the role of HMGB1 in DC maturation [31]. Recent work has clearly demonstrated the induction of inflammatory responses by extracellular histones from dying cells via TLR2 and TLR4 in acute kidney injury [32]. Taken together, these results indicate that the induction of Abreg against nuclear antigens such as histone H1 and HMGB1 must be one of the homeostatic phenomena that abrogate the inflammatory responses and may not be associated with any clinical manifestations.

The recurrence of autoimmune hepatitis or de novo autoimmune hepatitis has been characterized by the induction of auto-Abs [19, 20], but the precise roles of auto-Abs in the clinical manifestation of autoimmune hepatitis are still uncertain. Most researchers may speculate that the induction of de novo autoimmune hepatitis would accelerate the rejection response due to the synergistic effect of rejection and inflammatory responses. In this study, however, we observed the unique phenomenon that the transient induction of de novo autoimmune hepatitis by Con A injection paradoxically overcomes the rejection after OLT. However, despite prolonged survival of OLT (DA-LEW) with Con A injection, liver histology demonstrated that the massive infiltration of immune cells into the liver allografts was observed at day 7 after OLT (DA-LEW) with Con A injection, which was similar to that of liver graft of OLT (DA-LEW) without Con A injection. These results suggest that rejection with inflammatory responses in the early stage of Con A injection following OLT may play important roles for immune regulation. Similarly to our study, Li et al. recently reported the unique phenomenon that the acute rejection response after OLT was attenuated in the rats with hepatic alveolar echinococcosis infection, suggesting interference between the parasite infection and the rejection response [33]. We recently confirmed that the liver histology in the DA-PVG natural tolerance model also revealed a similar rejection pattern to the observations in the DA-LEW rejection combination at day 7 after OLT [34], suggesting that the involvement of the rejection and inflammatory responses is necessary to overcome rejection and subsequently induce tolerance without immunosuppressive drugs.

Additionally, Con A injection following OLT may lead the host immune system to a tolerogenic status with not only the suppression of DSA response but also the induction of Abreg against nuclear histone H1 and HMGB1. As we have mentioned in our recent review article [25], an initial mechanism for the induction of antinuclear Abs is the release of nuclear antigens, and the primary source of nuclear antigens would be damaged hepatic cells due to peritransplant ischemia/reperfusion injury, post-transplant rejection, and Con A-induced autoimmune hepatitis. Nuclear antigens including histone H1 and HMGB1 may act as “nuclear weapon” for induction of innate and adaptive immune responses. In this condition, the induction of corresponding auto-Abs may neutralize those nuclear antigens, resulting in the amelioration of inflammation and rejection. Thus, we speculate that the competitive balance between autoimmunity and alloimmunity is important for the prolongation of allograft survival. Another possibility for liver allograft acceptance is the activation of regulatory cells capable of suppressing proliferation of other cells by lectins [35, 36]. The prolonged allograft survival after transplantation by mitogenic lectins such as phytohemagglutinin, lentil lectin, and Con A has been reported in renal, skin, pancreas, and heart transplantation in mice, rats, and dogs [37], while it has not yet been explored in liver transplantation. However, in a murine model of Con A-induced autoimmune hepatitis, the mechanisms of Treg-mediated tolerance induction in response to liver inflammation are mentioned [1, 38, 39]. The putative mechanisms of lectin-induced allograft acceptance are not completely verified; however, we may explain possible immunosuppressive mechanisms by lectins through the induction of Abreg against nuclear histone H1 and HMGB1 which negatively regulates harmful T cell response in partly collaboration with Treg [40], resulting in the induction of allograft acceptance.

The induction of CD4^+^CD25^+^Foxp3^+^ Tregs, which primarily produce IL-10, is proposed as a possible mechanism for the protection and recovery from hepatic injury after Con A injection [1]. Furthermore, Fujii et al. reported the induction of autoantibody-producing B cells (i.e., B-1 cells) in the course of Con A-induced hepatic injury [41]. The therapeutic potential of naturally occurring IgM auto-Abs for prevention of autoimmune diabetes and for promotion of allograft survival has been recently reported [42]. B-1 cells are also known to produce the anti-inflammatory cytokine IL-10 [43]. Taking these results together, Con A injection after OLT may induce IL-10-producing Tregs and B-1 cells for the protection and recovery from acute rejection as well as autoimmune hepatitis. In this study, however, we have only confirmed the elevation of hepatic levels of Foxp3 and IL-10. Therefore, further studies including the hepatic and circulating levels of L-10-producing Tregs and B-1 cells should be performed to clarify the fundamental mechanisms of Tregs and B-1 cells in liver allograft acceptance.

In summary, our present data suggest that the transient induction of de novo autoimmune hepatitis by Con A injection after OLT may regulate the balance of autoimmunity and alloimmunity to overcome rejection and subsequently induce liver allograft acceptance. Although this study has revealed unique phenomena for prolongation of liver allograft survival, there are several limitations in this study including the transient induction of de novo autoimmune hepatitis by Con A injection, which may not completely mimic the clinical manifestation of de novo autoimmune hepatitis. In addition, much work remains to understand the mechanisms of immune modulation by the involvement of the Abreg against nuclear histone H1 and HMGB1 on Treg. We need to clarify the balance of autoimmunity and alloimmunity as well as the response to infectious diseases and liver inflammation in patients after liver transplantation to evaluate liver allograft rejection and acceptance.

## Abbreviations

Abreg: Regulatory antibody
Auto-Ab: Autoantibody
Con A: Concanavalin A
DA: Dark Agouti
DCs: Dendritic cells
DSA: Donor specific alloantibody
ELISA: Enzyme-linked immunosorbent assay
HMGB1: High-mobility group box 1
LEW: Lewis
MIP: Macrophage inflammatory protein
OLT: Orthotopic liver transplantation
PVG: Piebald Virol Glaxo
Tregs: Regulatory T cells.
